# Social determinants of health associated with psychological distress stratified by lifetime traumatic brain injury status and sex: Cross-sectional evidence from a population sample of adults in Ontario, Canada

**DOI:** 10.1371/journal.pone.0273072

**Published:** 2022-08-31

**Authors:** Vincy Chan, Lauren Marcus, Danielle Burlie, Robert E. Mann, Danielle Toccalino, Michael D. Cusimano, Gabriela Ilie, Angela Colantonio

**Affiliations:** 1 KITE-Toronto Rehabilitation Institute, University Health Network, Toronto, Ontario, Canada; 2 Institute of Health Policy, Management and Evaluation, University of Toronto, Toronto, Ontario, Canada; 3 Temerty Faculty of Medicine, Rehabilitation Sciences Institute, University of Toronto, Toronto, Ontario, Canada; 4 Temerty Faculty of Medicine, Department of Occupational Science & Occupational Therapy, University of Toronto, Toronto, Ontario, Canada; 5 Centre for Addiction and Mental Health, Toronto, Ontario, Canada; 6 Dalla Lana School of Public Health, University of Toronto, Toronto, Ontario, Canada; 7 St. Michael’s Hospital, Unity Health Toronto, Toronto, Ontario, Canada; 8 Division of Neurosurgery, Department of Surgery, University of Toronto, Toronto, Ontario, Canada; 9 Department of Community Health and Epidemiology, Dalhousie University, Halifax, Nova Scotia; University of Otago, NEW ZEALAND

## Abstract

This study identified the social determinants of health (SDoH) associated with psychological distress in adults with and without a self-reported history of traumatic brain injury (TBI), stratified by sex. Data from the 2014–2017 cycles of the Centre for Addiction and Mental Health Monitor Survey, a representative survey of adults ≥18 years in Ontario, Canada, were analyzed (N = 7,214). The six-item version of the Kessler Psychological Distress Scale was used to determine moderate to severe psychological distress. Self-reported lifetime TBI was defined as a head injury resulting in a loss of consciousness for ≥5 minutes or at least one-night stay in the hospital (16.4%). Among individuals reporting a history of TBI, 30.2% of males and 40.1% of females reported psychological distress (p = 0.0109). Among individuals who did not report a history of TBI, 17.9% of males and 23.5% of females reported psychological distress (p<0.0001). Multivariable logistic regression analyses showed that the SDoH significantly associated with elevated psychological distress were similar between individuals with and without a history of TBI. This included unemployment, student, or ‘other’ employment status among both males and females; income below the provincial median and age 65+ among males; and rural residence among females. This study highlighted opportunities for targeted population-level interventions, namely accessible and affordable mental health supports for individuals with lower income. Notably, this study presented evidence suggesting adaptations to existing services to accommodate challenges associated with TBI should be explored, given the finite and competing demands for mental health care and resources.

## Introduction

Traumatic brain injury (TBI) has been defined as “an alteration in brain function, or other evidence of brain pathology, caused by an external force” [1]. It is a global public health concern and it is estimated that 50% of the world’s population will experience a TBI in their lifetime [2]. Even a mild TBI (mTBI) can affect every domain of functioning, including an increased risk for adverse mental health [3,4]. In fact, mental health challenges are common among individuals with TBI [5,6]. For example, a recent prospective longitudinal study on patients with mTBI in the emergency department found that 21% of patients had a history of psychiatric disorders and almost 10% of patients experienced depression at 3- and 6-months post-mTBI [7]. Surveillance data on adults with a history of TBI with loss of consciousness (LOC) found that these individuals had higher odds of lifetime depression compared to adults without a lifetime history of TBI with LOC [8]. Furthermore, the presence of mental health disorders are associated with adverse health and health system-level outcomes. For example, research on individuals who received medical attention for a TBI found that those with comorbid mental health disorders were more likely to experience re-hospitalizations, delayed discharge, increased direct medical costs post-TBI, and reduced functional gain during inpatient rehabilitation [9–12].

Unfortunately, gaps in healthcare and supports across the continuum of care persist for individuals with TBI who also experience mental health challenges [13]. This is problematic because early intervention and access to mental health services and supports are critical to prevent poor mental health and adverse TBI-related outcomes post-injury [6]. In planning health services and supports, it is critical to consider the social determinants of health (SDoH) to promote comprehensive access to care and prevent health inequities [14]. SDoH are social and economic factors that can positively or negatively influence both individual and population health and are globally recognized as some of the most important factors impacting health [15]. For individuals with TBI, the consideration of SDoH in healthcare planning is particularly important because TBI is disproportionately prevalent among individuals with unfavourable SDoH such as unemployment, lower levels of education, and low socioeconomic status [16,17]. Furthermore, a recent report by the Public Health Agency of Canada identified individuals living with disability as one of the populations who experience significant health inequities (others included Indigenous people, sexual and racial minorities, immigrants, individuals with lower income and education level, and those who experience unemployment) [18]. Concurrently, it is well established that adverse SDoH are barriers to accessing mental health care [19,20]. SDoH associated with mental health challenges in population samples that capture individuals with milder TBIs who may not require medical attention must be identified to inform opportunities for targeted public health interventions, as targeted interventions along-side universal policies/interventions has been identified as an actionable practice to advance health equity in Canada [18]. Given the finite and competing demands for mental health care and resources, identifying similar SDoH between individuals with and without TBI may inform opportunities to adapt existing population-level services and supports for individuals with TBI.

The objectives of this study were to determine the prevalence of psychological distress and to identify the SDoH associated with psychological distress in a population sample of adults ≥18 years of age. This study stratified the sample by self-reported lifetime TBI status and sex to create four mutually exclusive subgroups–(1) males with a lifetime history of TBI, (2) females with a lifetime history of TBI, (3) males without a lifetime history of TBI, and (4) females without a lifetime history of TBI. The stratification of the sample by TBI status enabled us to determine similar and different SDoH associated with psychological distress to inform opportunities to adapt existing population-level mental health services and supports for individuals with TBI. The stratification of the sample by sex enabled us to account for the interaction of sex with SDoH and to identify SDoH associated with psychological distress specifically among males and females to inform sex-sensitive interventions. This stratification is particularly important because sex and gender differences in mental health [21–23] and in outcomes post-TBI have been reported in both the general population and in specific sub-populations [10–12,22,24–27]. However, we also acknowledge research studies on TBI and mental health that have reported no sex and/or gender differences; for example, in one study, the reporting of mild depression did not differ between men and women at one year post-TBI [28]. Similarly, population-based studies that assessed the impact of pre-existing mental health disorders found that among both males and females, a history of mental health disorders significantly reduced functional outcome and increased direct medical cost post-injury [11,12]. Similarly, pre-injury mental health was significantly associated with excess mortality among both male and female patients who experienced a mild or severe TBI [29]. The current lack of research explicitly considering sex is a significant limitation that must be addressed, as it is a major barrier to targeted prevention and support for males and females with TBI and mental health challenges [30–32].

## Methods

This study was approved by the Research Ethics Boards (REB) of the Centre for Addiction and Mental Health (CAMH), University of Toronto, and York University. The reporting of this study followed the Strengthening of the Reporting of Observational Studies in Epidemiology [33] and Sex and Gender Equity in Research guidelines [32].

### Sample

Data on participants in the 2014 to 2017 cycles of the CAMH Monitor Surveys were included. The CAMH Monitor is a telephone survey (landlines and cellphones) of Ontario adults ≥18 years of age administered by the Institute for Social Research at York University and is the longest ongoing (since 1977) population survey of mental health and addictions in Ontario, Canada [34]. A two-stage probability selection procedure (household, respondent) using random-digit-dialing methods and Computer Assisted Telephone Interviewing were employed [34]. The response rates were 45% in 2014, 41% in 2015, 38% in 2016, and 35% in 2017 [34]. Informed verbal consent was approved by REB and was obtained from study participants prior to administering the survey; this information was stored in a password-protected computer.

### Variables

Lifetime TBI was self-reported based on a definition of a head injury resulting in a loss of consciousness (LOC) (“knocked out”) for at least 5 minutes or at least one night stay in the hospital. Specifically, participants were asked “How many times, if ever in your life, have you had a head injury like this?” [34]. Individuals who self-reported experiencing at least one head injury were determined to have experienced a TBI in their lifetime. This definition is consistent with operational definitions of TBI by Menon and colleagues: “an alteration in brain function (defined as any period of loss of, or decreased, consciousness; any loss of memory for events immediately before or after the injury; neurological deficits; or any alteration in mental state at the time of injury), or other evidence of brain pathology, caused by an external force” [1] and the Diagnostic and Statistical Manual of Mental Disorders Fifth Edition (DSM-V): “an impact to the head or other mechanisms of rapid movement or displacement of the brain within the skull, with one or more of the following: LOC; posttraumatic amnesia; disorientation and confusion; or neurological signs” [35].

The main variable of interest was moderate to severe psychological distress, measured using the six-item version of the Kessler Psychological Distress Scale [(K6); score of 5–12: moderate, score of 13+: serious]. The K6 is a reliable and valid tool to screen for mood and anxiety disorders [36–38] across race/culture, gender, and other sub-populations [39–46]. The Cronbach’s reliability-coefficient for the six items in this sample was 0.77.

Social, demographic, and economic variables in the CAMH Monitor were categorized into SDoH as listed by the Government of Canada: [15] (1) income and social status: household income [<$70,000 and ≥$70,000, determined based on the median provincial household income in Ontario ($74,287)]; [47] (2) employment and working conditions: employment status (employed, unemployed, retired, student, and other); (3) education and literacy: education (less than high school, completed high school, some post-secondary, technical/college/bachelor’s degree, and postgraduate/professional degree); (4) access to health services: rurality (rural and non-rural), as there is evidence of disparities in access to services across the rural-urban continuum; [48] (5) social supports and coping skills: marital status (married/living with partner, widowed, divorced/separated, and never married), as there is evidence that marital status influences level of perceived social support; [49] (6) biology & genetic endowment: age (18–29, 30–39, 40–49, 50–64, and 65+ years); (7) culture: race (white and racial minority), immigration status (born in Canada and born outside of Canada), and language spoken at home (English and non-English); and (8) sex: sex (male and female) and sexual orientation (heterosexual and non-heterosexual).

### Statistical analyses

The sample was stratified by sex and self-reported lifetime TBI status to create four mutually exclusive subgroups–(1) males who reported experiencing a lifetime TBI (herein referred to as ‘males with TBI’, (2) females who reported experiencing a lifetime TBI (females with TBI), (3) males who did not report experiencing a lifetime TBI (males without TBI), and (4) females who did not report experiencing a lifetime TBI (females without TBI). To account for the complex survey data, analyses were conducted using the Taylor Series Linearization in SAS v9.4. The prevalence of moderate to severe psychological distress was determined for the four subgroups and second-order Rao-Scott adjusted chi-square tests were conducted to assess the prevalence of psychological distress between sexes and TBI status. Multivariable logistic regression analyses were conducted to identify SDoH associated with psychological distress for the four subgroups. The final analyses were based on a design with 6 strata (region) and 7,214 participants. All data presented in this study were based on valid responses; participants who responded “don’t know/refused to answer” or that had missing data were excluded from the analyses. Furthermore, the data reported are based on the weighted sample size and are therefore considered representative for the adult population of Ontario.

## Results

Among 7,214 participants in the 2014 to 2017 cycles of the CAMH Monitor, 16.4% (95% Confidence Interval [CI]: 15.3%-17.5%) reported experiencing a TBI (N = 1,185). Within this group, 30.2% (95% CI: 25.3%-35.2%) of males and 40.1% (95% CI: 34.4%-46.0%) of females reported psychological distress (p = 0.0109). Among individuals who did not report experiencing a TBI (N = 6,029), 17.9% (95% CI: 15.8%-20.1%) of males and 23.5% (21.7%-25.3%) of females reported psychological distress (p<0.0001). The prevalence of psychological distress was significantly higher among individuals with TBI than those without TBI (males: p<0.0001, females: p<0.0001).

Among males with TBI, the SDoH significantly associated with psychological distress included (1) household income: <$70,000 vs. ≥$70,000 (OR = 2.896, 95% CI: 1.579–5.312); (2) employment status: unemployed (OR = 4.497, 95% CI: 1.372–14.742), student (OR = 5.464, 95% CI: 1.281–23.318), and other (OR = 13.894, 95% CI: 4.474–43.149) vs. employed; (3) marital status: divorced/separated vs. married/living with partner (OR = 2.332, 95% CI: 1.133–4.800); and (4) age ≥65 vs. 50–64 years (OR = 0.330, 95% CI: 0.162–0.674).

Among males without TBI, the SDoH significantly associated with psychological distress included (1) household income: <$70,000 vs. ≥$70,000 (OR = 1.449, 95% CI: 1.020–2.057); (2) employment status: unemployed (OR = 4.157, 95% CI: 2.022–8.548), student (OR = 2.236, 95% CI: 1.107–4.519), and other (OR = 4.443, 95% CI: 2.091–9.443) vs. employed; (3) education: post-graduate/professional degree vs. technical/college/bachelor’s degree (OR = 0.569, 95% CI: 0.338–0.956); (4) marital status: widowed vs. married/living with partner (OR = 1.721; 95% CI: 1.009–2.938); (5) age 30–39 vs. 50–64 years (OR = 1.990; 95% CI: 1.271–3.116); and (6) sexual orientation: non-heterosexual vs. heterosexual (OR = 2.189, 95% CI: 1.104–4.339).

Among females with TBI, the SDoH significantly associated with psychological distress included: (1) employment status: other vs. employed (OR = 5.765, 95% CI: 1.669–19.559); (2) education: <high school vs. technical/college/bachelor’s degree (OR = 2.829, 95% CI: 1.187–6.747), post-graduate/professional degree vs. technical/college/bachelor’s degree (OR = 0.414, 95% CI: 0.173–0.991); and (3) rurality: rural vs. non-rural (OR = 2.204, 95% CI: 1.116–4.354).

Among females without TBI, the SDoH significantly associated with psychological distress included: (1) household income: <$70,000 vs. ≥$70,000 (OR = 1.452: 1.132–1.863); (2) employment status: other vs. employed (OR = 2.699, 95% CI: 1.536–4.742); (3) rurality: rural vs. non-rural (OR = 0.602; 95% CI: 0.443–0.819); (4) age ≥65 vs. 50–64 years (OR = 0.620, 95% CI: 0.448–0.860); (5) sexual orientation: non-heterosexual vs. heterosexual (OR = 2.753; 95% CI: 1.567–4.838).

Table 1 presents a visual comparison of SDoH significantly associated with psychological distress among the four subgroups. Tables 2 and 3 present the prevalence of psychological distress and results from the multivariable logistic regressions of SDoH and psychological distress among males and females, respectively.

**Table 1:** Visual comparison of the SDoH associated with psychological distress, by sex and TBI status, from multivariable logistic regression models.

Social Determinant of Health	TBI		No TBI	
	Males	Females	Males	Females
**Income & Social Status**				
**Household income**				
<$70,000	↑	-	↑	↑
$70,000+	Ref	Ref	Ref	Ref
**Employment & Working Conditions**				
**Employment status**				
Employed	Ref	Ref	Ref	Ref
Unemployed	↑	-	↑	-
Retired	-	-	-	-
Student	↑	-	↑	-
Other	↑	↑	↑	↑
**Education & Literacy**				
**Education**				
Less than high school	-	↑	-	-
Completed high school	-	-	-	-
Some post-secondary	-	-	-	-
Technical/college/bachelor’s degree	Ref	Ref	Ref	Ref
Postgraduate/professional degree	-	↓	↓	-
**Access to Health Services**				
**Rurality**				
Rural	-	↑	-	↓
Non-rural	Ref	Ref	Ref	Ref
**Social Supports & Coping Skills**				
**Marital Status**				
Married/living with partner	Ref	Ref	Ref	Ref
Widowed	-	-	↑	-
Divorced/separated	↑	-	-	-
Never married	-	-	-	-
**Biology & Genetic Endowment**				
**Age**				
18–29	-	-	-	-
30–39	-	-	↑	-
40–49	-	-	-	-
50–64	Ref	Ref	Ref	Ref
65+	↓	-	-	↓
**Sex/Gender**				
**Sexual orientation**				
Heterosexual	Ref	Ref	Ref	Ref
Non-heterosexual	-	-	↑	↑
**Culture**				
**Race**				
White	Ref	Ref	Ref	Ref
Racial minority	-	-	-	-
**Immigrant status**				
Born in Canada	Ref	Ref	Ref	Ref
Born outside Canada	-	-	-	-
**Language spoken at home**				
English	Ref	Ref	Ref	Ref
Non-English	-	-	-	-

**Ref:** Reference; **SDoH:** Social determinants of health; **TBI:** Traumatic brain injury.

↑ = Significantly associated with increased odds of reporting psychological distress.

↓ = Significantly associated with decreased odds of reporting psychological distress.

- = Not statistically significant in multivariable logistic regression model.

**Table 2:** Prevalence of psychological distress and multivariable logistic regression analyses of SDoH and psychological distress among males with and without TBI.

Social Determinants of Health	Males with TBI					Males without TBI				
	Total Sample	Psychological Distress	Multivariable Logistic Regression			Total Sample	Psychological Distress	Multivariable Logistic Regression		
	N	% Yes	OR	95% CI		N	% Yes	OR	95% CI	
**Total**	754	30.2				2799	17.9			
**Income & Social Status**										
**Household income**										
<$70,000	223	49.8	2.896	1.579	5.312	780	23.5	1.449	1.02	2.057
$70,000+	531	22.0	1.000			2019	15.8	1.000		
**Employment & Working Conditions**										
**Employment status**										
Employed	490	21.4	1.000			1931	15.8	1.000		
Unemployed	36	58.3	4.497	1.372	14.742	88	53.4	4.157	2.022	8.548
Retired	142	22.5	1.827	0.939	3.554	573	9.6	0.713	0.45	1.132
Student	49	77.6	5.465	1.281	23.318	158	44.3	2.236	1.107	4.519
Other	37	86.5	13.894	4.474	43.149	49	49.0	4.443	2.091	9.443
**Education & Literacy**										
**Education**										
Less than high school	67	46.3	0.959	0.421	2.183	154	15.6	0.991	0.533	1.841
Completed high school	155	31.0	0.690	0.343	1.390	583	21.6	1.145	0.775	1.691
Some post-secondary	112	38.4	0.896	0.393	2.044	262	30.9	1.598	0.951	2.684
Technical/college/bachelor’s degree	347	27.7	1.000			1396	16.6	1.000		
Postgraduate/professional degree	73	13.7	0.575	0.221	1.492	404	9.4	0.569	0.338	0.956
**Physical Environments and Access to Health Services**										
**Rurality**										
Rural	110	29.1	1.017	0.488	2.119	367	12.8	0.747	0.493	1.130
Non-rural	644	30.4	1.000			2432	18.7	1.000		
**Social Supports & Coping Skills**										
**Marital Status**										
Married/living with partner	506	20.8	1.000			1961	12.9	1.000		
Widowed	20	55.0	2.698	0.944	7.710	61	16.4	1.721	1.009	2.938
Divorced/separated	60	45.0	2.332	1.133	4.800	113	17.7	1.314	0.815	2.119
Never married	168	50.6	1.361	0.533	3.476	664	33.0	1.568	1.000	2.457
**Biology & Genetic Endowment**										
**Age**										
18–29	133	58.6	3.137	0.962	10.228	524	32.8	1.678	0.940	2.996
30–39	96	37.5	1.671	0.721	3.871	483	21.9	1.990	1.271	3.116
40–49	149	19.5	1.150	0.554	2.387	510	14.9	1.374	0.873	2.162
50–64	262	25.2	1.000			802	12.0	1.000		
65+	114	16.7	0.330	0.162	0.674	480	10.6	1.092	0.684	1.746
**Gender**										
**Sexual orientation**										
Heterosexual	734	29.6	1.000			2710	17.3	1.000		
Non-heterosexual	20	55.0	0.826	0.166	4.121	89	34.8	2.189	1.104	4.339
**Culture**										
**Race**										
White	668	30.1	1.000			2286	16.6	1.000		
Racial minority	86	31.4	0.766	0.323	1.816	513	23.6	1.259	0.792	2.001
**Immigrant status**										
Born in Canada	643	30.8	1.000			2184	18.4	1.000		
Born outside Canada	111	27.0	1.265	0.603	2.654	615	16.3	1.086	0.693	1.701
**Language spoken at home**										
English	714	30.0	1.000			2499	17.9	1.000		
Non-English	40	35.0	1.925	0.581	6.386	300	17.7	0.754	0.438	1.298

**CI:** Confidence Interval; **OR:** Odds ratio; **TBI:** Traumatic brain injury.

**Table 3:** Prevalence of psychological distress and multivariable logistic regression analyses of SDoH and psychological distress among females with and without TBI.

	Females with TBI					Females without TBI				
	Total Sample	Psychological Distress	Multivariable Logistic Regression			Total Sample	Psychological Distress	Multivariable Logistic Regression		
	N	% Yes	OR	95% CI		N	% Yes	OR	95% CI	
**Total**	431	40.1				3230	23.5			
**Income & Social Status**										
**Household income**										
<$70,000	172	40.7	0.873	0.462	1.650	1066	28.1	1.452	1.132	1.863
$70,000+	259	39.8	1.000			2164	21.3	1.000		
**Employment & Working Conditions**										
**Employment status**										
Employed	246	41.9	1.000			2031	22.5	1.000		
Unemployed	55	36.4	0.668	0.269	1.660	251	23.1	0.960	0.656	1.404
Retired	89	21.3	0.719	0.375	1.379	634	17.0	0.920	0.674	1.256
Student	15	73.3	2.403	0.454	12.704	239	42.3	1.345	0.762	2.376
Other	26	76.9	5.765	1.699	19.559	75	48.0	2.699	1.536	4.742
**Education & Literacy**										
**Education**										
Less than high school	24	58.3	2.829	1.187	6.747	132	26.5	1.462	0.972	2.201
Completed high school	104	43.3	1.569	0.789	3.120	567	25.9	1.089	0.817	1.451
Some post-secondary	53	43.4	1.218	0.543	2.732	341	29.9	1.137	0.793	1.630
Technical/college/bachelor’s degree	204	39.7	1.000			1778	21.8	1.000		
Postgraduate/professional degree	46	21.7	0.414	0.173	0.991	412	21.6	0.993	0.725	1.360
**Physical Environments and Access to Health Services**										
**Rurality**										
Rural	62	51.6	2.204	1.116	4.354	378	15.9	0.602	0.443	0.819
Non-rural	369	38.2	1.000			2852	24.5	1.000		
**Social Supports & Coping Skills**										
**Marital Status**										
Married/living with partner	274	37.6	1.000			2176	20.1	1.000		
Widowed	32	28.1	1.151	0.486	2.722	192	18.8	1.050	0.715	1.541
Divorced/separated	44	45.5	1.372	0.676	2.786	225	27.1	1.292	0.948	1.761
Never married	81	50.6	0.929	0.345	2.504	637	35.5	1.212	0.830	1.770
**Biology & Genetic Endowment**										
**Age**										
18–29	61	59.0	2.592	0.791	8.499	518	37.3	1.579	0.986	2.530
30–39	63	46.0	2.041	0.875	4.761	542	24.4	1.176	0.845	1.636
40–49	93	48.4	1.921	0.952	3.879	702	21.7	1.079	0.814	1.431
50–64	135	34.8	1.000			893	21.5	1.000		
65+	79	20.3	0.616	0.320	1.187	575	15.8	0.620	0.448	0.860
**Gender**										
**Sexual orientation**										
Heterosexual	410	38.5	1.000			3151	22.8	1.000		
Non-heterosexual	21	71.4	2.784	0.850	9.119	79	53.2	2.753	1.567	4.838
**Culture**										
**Race**										
White	386	39.9	1.000			2681	22.4	1.000		
Racial minority	45	42.2	1.065	0.445	2.550	549	29.0	0.884	0.614	1.271
**Immigrant status**										
Born in Canada	341	41.1	1.000			2493	22.9	1.000		
Born outside Canada	90	36.7	1.217	0.660	2.244	737	25.6	1.125	0.841	1.506
**Language spoken at home**										
English	403	40.0	1.000			2823	22.7	1.000		
Non-English	28	42.9	0.955	0.331	2.753	407	29.5	1.221	0.861	1.731

**CI:** Confidence Interval; **OR:** Odds ratio; **TBI:** Traumatic brain injury.

## Discussion

This study identified SDoH associated with psychological distress among Ontarian adults, by sex and self-reported TBI status. Several key findings were identified from this study. First, psychological distress is prevalent among Ontarian adults, particularly among individuals with TBI. Second, SDoH significantly associated with psychological distress were similar between individuals with and without a self-reported lifetime history of TBI. Third, specifically among individuals who reported experiencing a TBI, the SDoH associated with psychological distress differed by sex.

In this population sample of Ontario adults, 30.2% of males and 40.1% of females with TBI, and 17.9% of males and 23.5% of females without TBI, reported experiencing moderate to serious psychological distress. The prevalence of psychological distress was significantly higher among individuals with TBI and, regardless of TBI status, the prevalence of psychological distress was higher among females compared to males. First, this sex difference in the reporting of psychological distress is not surprising, as it is well-documented that anxiety and depression are more common among females [21–23]. We acknowledge that this study assessed self-reported psychological distress; as such, this finding may also reflect differences observed in the reporting of health challenges among males and females [50]. However, in this population sample representative of Ontario adults, an alarmingly high prevalence of psychological distress was reported among both males and females with TBI. This highlights the urgent need to screen for mental health challenges among individuals with TBI to prevent poor prognosis and enable post-injury care, including rehabilitation, and to consider the potential impact of psychological distress on treatment [6]. This current study did not include the provision of mental health or TBI resources to participants at the end of the survey (e.g., hotline or websites of mental health or brain injury supports services); future research, including phone surveys such as the CAMH Monitor, are encouraged to consider the feasibility of providing such resources at the end of the survey to maximize the availability of support for individuals with experiences of psychological distress and/or TBI. We also acknowledge that another variable captured in the SDoH of sex was sexual orientation, which was significantly associated with increased odds of reporting psychological distress only among individuals without TBI. However, this finding should be interpreted with caution given the smaller sample of individuals identifying as non-heterosexual.

Overall, the SDoH associated with psychological distress identified in this study suggests that accessible and affordable mental health supports for individuals with lower income must be available. Furthermore, findings from the multivariable logistic regression analyses of the four subgroups showed that the SDoH associated with psychological distress were similar among adults with and without TBI. This included employment (unemployed, student status, and other employment) among both males and females; income below the provincial median and age 65+ among males; and rural residence among females. This suggests that opportunities to adapt existing population-level mental health services and supports for individuals with TBI should be explored, particularly those that are already targeting individuals experiencing these adverse SDoH. This approach is consistent with a report from the Public Health Agency of Canada, which lists “deploy[ing] a combination of targeted interventions and universal policies/ interventions” as one of the key actions to advance health equity within the Canadian context [18]. For TBI, this may include accommodations at the level of acute care and rehabilitation care to address cognitive challenge experienced by individuals with TBI. These accommodations can address potential barriers associated with participation and attendance and may include reminder calls or alerts to attend appointments or sessions or using a ‘teach-back’ method to check for understanding [30,51–53]. Furthermore, education on TBI for healthcare professionals within mental health settings should be explored to enable them to recognize clients with TBI so delays in treatments may be prevented [54] and existing services may be adapted to address unique challenges associated with TBI [30].

Specifically, among individuals who reported experiencing a lifetime TBI, the SDoH associated with psychological distress differed by sex. Income, marital status, and age were significant determinants among males only, while education and rural residence were significant among females only. First, this finding complements the growing literature on sex differences as predictors of and influencing factors in health and health outcomes among individuals with TBI [10–12,21,22,24–27] and reiterates the importance of integrating sex and gender considerations in TBI research [30–33]. Second, while we stratified the data by sex, the influence of gender cannot be dismissed. For example, gender, income, employment, and education are inter-related; research has shown that women are significantly less likely to be engaged in full-time work and have significantly lower average pre-injury income [55–57]. Concurrently, education is correlated with income and employment opportunities [58], while pay gaps between men and women as well as women-dominated vs. men-dominated occupations exist [59]. Finally, we acknowledge this study accounted for the interaction of sex with SDoH through sex-stratified analyses. Thus, this study identified the SDoH significantly associated with psychological distress by sex, and not sex differences in the magnitude of the associations. Research to determine the extent to which males and females with specified SDoH experience psychological distress differently should be conducted to further inform interventions that are sensitive to the unique needs of males and females. Overall, research must continue to consider the intersection of sex, gender, and SDoH in design, analysis, and interpretation to support best practices and science [30–33].

### Limitations and strengths

A key limitation is that individuals without access to a landline or cellphone [34] or who are institutionalized are excluded from this study. In addition, participants who consented to participate in the CAMH Monitor may represent a different population in terms of experiences of SDoH and psychological distress, compared to participants who did not provide consent to participate. We are unable to assess the impact of this non-response bias in our study. We also recognize that individuals with more severe TBI or cognitive deficits who may have difficulty following the questions of the survey or those experiencing unfavourable SDoH may also be under-represented in CAMH Monitor participants. However, among participants of this survey, there was no significant difference between individuals with and without TBI who reported the survey as difficult (7.4% and 6.9%, respectively, p = 0.8798). Second, individuals who cannot speak or understand English were excluded from participation as a translator/translated survey was not available; this may explain the small sample of individuals in the survey who reported speaking non-English language at home and the lack of statistically significant findings on the SDoH of culture. Third, the screening question used to detect self-reported TBI may miss individuals with mild TBIs who did not require a one-night stay in the hospital or experience a LOC for at least 5 minutes. As such, the sample of individuals with TBI is likely underestimated and the findings may not be applicable to the experiences of all individuals who experience a TBI across the continuum of severity. Importantly, the CAMH Monitor does not include information on the characteristics of the TBI, such as mechanism of injury or injury severity. This is a limitation, as outcomes after TBI often depends on both the quality of healthcare provided as well as injury characteristics, such as cause of injury and injury severity [2]. Finally, we also acknowledge that the CAMH Monitor does not capture all SDoH and none of the categories of SDoH identified in this study are comprehensively described. Risk of recall and non-response bias are also present, as only valid responses were included and data were self-reported. However, self-reported TBI is considered the gold standard to comprehensively identify lifetime TBI in research, [60] and the K6 is a valid and reliable tool to screen for psychological distress [36–38]. Finally, we acknowledge that associations identified in this study may be bi-directional in nature and causal inferences cannot be made from cross-sectional surveys.

Despite these limitations, a key strength of this study is the large overall sample size and that the CAMH Monitor captures a geographically representative sample of Ontarian adults. The screening of TBI at the population level also captured individuals with TBI who may not seek medical attention for their TBI, as it has been noted that relying on medical records is insufficient to detect lifetime history of TBI [60,61]. As current estimates of TBI and psychological distress primarily rely on health system level data that only capture TBI and mental health seen in healthcare settings, this study is a valuable addition to the literature. Finally, this study stratified the sample by sex to account for the interaction of sex with SDoH and to identify the SDoH associated with psychological distress specifically among males and females to inform sex-sensitive interventions.

## Conclusion

Psychological distress is prevalent among Ontarian adults, particularly among those with TBI; 30.2% of males and 40.1% of females with TBI and 17.9% of males and 23.5% of females without TBI reported experiencing moderate to severe psychological distress. This study highlights the need to identify and address psychological distress, particularly in those with TBI, so post-injury care can consider the potential impact of psychological distress on treatment choice, adherence, and outcomes. The SDoH associated with psychological distress among individuals with and without TBI were similar, suggesting that adaptations to existing population-level mental health services and supports may be opportunities to support individuals with TBI, given the finite and competing demands of mental health care resources. Finally, among individuals with TBI, the SDoH associated with psychological distress differed by sex, further highlighting the importance of considering the intersection of sex, gender, and SDoH in research design, analysis, and interpretation of findings to inform interventions that are sensitive to the unique needs of males and females.

## Abbreviations

CAMH: Centre for Addiction and Mental Health
CI: Confidence Interval
K6: Kessler Psychological Distress Scale, Six-Item Version
mTBI: Mild traumatic brain injury
OR: Odds ratio
SDoH: Social determinants of health
TBI: Traumatic brain injury
